# The Artificial Intelligence System for the Generation of Sports Education Guidance Model and Physical Fitness Evaluation Under Deep Learning

**DOI:** 10.3389/fpubh.2022.917053

**Published:** 2022-08-09

**Authors:** Yuanqing Li, Xiangliang Li

**Affiliations:** ^1^School of Physical Education, Huanghuai University, Zhumadian, China; ^2^Life Education Research Center, Henan University, Kaifeng, China; ^3^Physical Education and Health Department, Shanghai Lixin University of Accounting and Finance, Shanghai, China

**Keywords:** artificial intelligence, physical fitness evaluation, cognition, sports education guidance, deep learning

## Abstract

In recent years, China's achievements in artificial intelligence (AI) have attracted the attention of the world, and AI technology has penetrated into all walks of life. In particular, the in-depth integration of AI technology with sports education guidance and physical fitness evaluation has achieved very significant progress and results, which has improved the quality of life of people and provided more high-quality, customized, and personalized health management services for human beings. This study aimed to explore the application model of deep learning in sports education and guidance and in the analysis of the residents' physical fitness, so as to formulate a personalized and intelligent exercise program. The residents of A and B units are selected as the research object to evaluate the physical fitness. Subsequently, the self-designed questionnaire is used to survey the chronic disease online, and the acquired data are put into a deep learning model for the analysis to obtain the physique scoring results and exercise guidance. According to the results of physical fitness evaluation, the proportion of overweight was the highest (40.4%), followed by fatty liver (24.3%) and hyperlipidemia (20.4%), showing high incidence in people aged 41–50 years. The highest incidence of female gynecological diseases was gout (23.0%) and hyperlipidemia (20.6%). After exercise therapy, the scores were excellent and good. Conclusions: The database SQL Server 2005 was a platform for storing all kinds of data and knowledge-based rule information. The user's access service was provided by the remote server via the browser. Therefore, building a rule-based reasoning mechanism can realize physical test data collection, physical fitness evaluation, and information management for improving physical fitness.

## Introduction

Physique is the material basis of life activities and labor of the human body. It is a comprehensive, developed, and relatively stable function based on inheritance and acquisition of clear human morphological structure, physical functions, psychological factors, and adaptability (1, 2). In addition, it is necessary to mention other areas of research related to physical anthropology of science. Anthropology mainly studies the formation and distribution of human physical characteristics in the process of human origin and evolution. Human physical characteristics and social relations are also the focuses of anthropological research, including other aspects of human structure and function, morphology, genetics, and variation (3). In this study, the scope of physical fitness investigation is enriched, and the prospects of physical research on physical changes and development laws are expanded at various stages. Studies have shown that personal, age, and social factors can exert the effects on residents of different nationalities and modern physique (4–10).

Exercise prescription is derived from medical prescriptions, originally proposed by Kapochic (an American physiologist in the 1950's), and the term “exercise prescription” was formally used by the Japanese scholar Michio Inaaki in 1960 (11). In 1969, the World Health Organization (WHO) officially promoted the use of the term “exercise prescription.” “Exercise prescription” is based on health check, physical examination, and physical examination and is combined with the characteristics of the living environment and sports interests. It should be noted that the prescription content suitable for the individual includes time, frequency, and exercise mode. Individuals can regularly plan the exercises to achieve the fitness and disease treatment (12, 13).

In recent years, artificial intelligence (AI) industry in China has developed rapidly, which has also promoted the crazy growth of the AI market and enterprises. AI technology has been applied to about 20 industries such as business centers, hotels, banks, art galleries, hospitals, and education and is suitable for all fields of future society (14–17). At present, smart bracelets, smart sports shoes, smart glasses, smart shirts, and other wearable smart devices in the sports and fitness industries have become indispensable sports products for residents. They can monitor the health data of users in real time so that residents can correctly understand their own health status, fitness methods, and fitness effects, so as to prevent, intervene, and provide services to improve the health status of students (18). The pace, calories, speed, and heart rate are monitored to formulate the most suitable fitness management service plan for the exercise intensity. These smart products will inevitably lead personal sports and health management services to meet individual needs and provide convenient conditions (19). Based on the basic theory of decision support system (DSS), the asp Network Model-View-Controller (MVC) development framework, Visual Studio 2008 development environment, and structured query language (SQL) Server 200Counter5 are adopted in this study to realize the intelligent exercise prescription system and promote the health of citizens with the support of deep learning.

In this study, a comprehensive evaluation model of individual physical fitness is established, a physical health evaluation system based on the system structure is constructed and implemented, the residents of A and B units as the research objects are investigated, and the physical fitness of the residents are evaluated. Subsequently, the self-designed questionnaire is applied to conduct chronic disease surveys in the form of online questionnaires, and the acquired data are put into a deep learning model for analysis to give the physique scoring results and exercise guidance. The back propagation neural networks (BPNNs) algorithm is introduced to comprehensively evaluate the physical health of the human body, which solves the shortcoming that the upper and lower values of some index critical points are not much different when the past evaluation standards are promoted and used in a large area.

## Methods

### Selection of Research Objects

**Figure 1:**
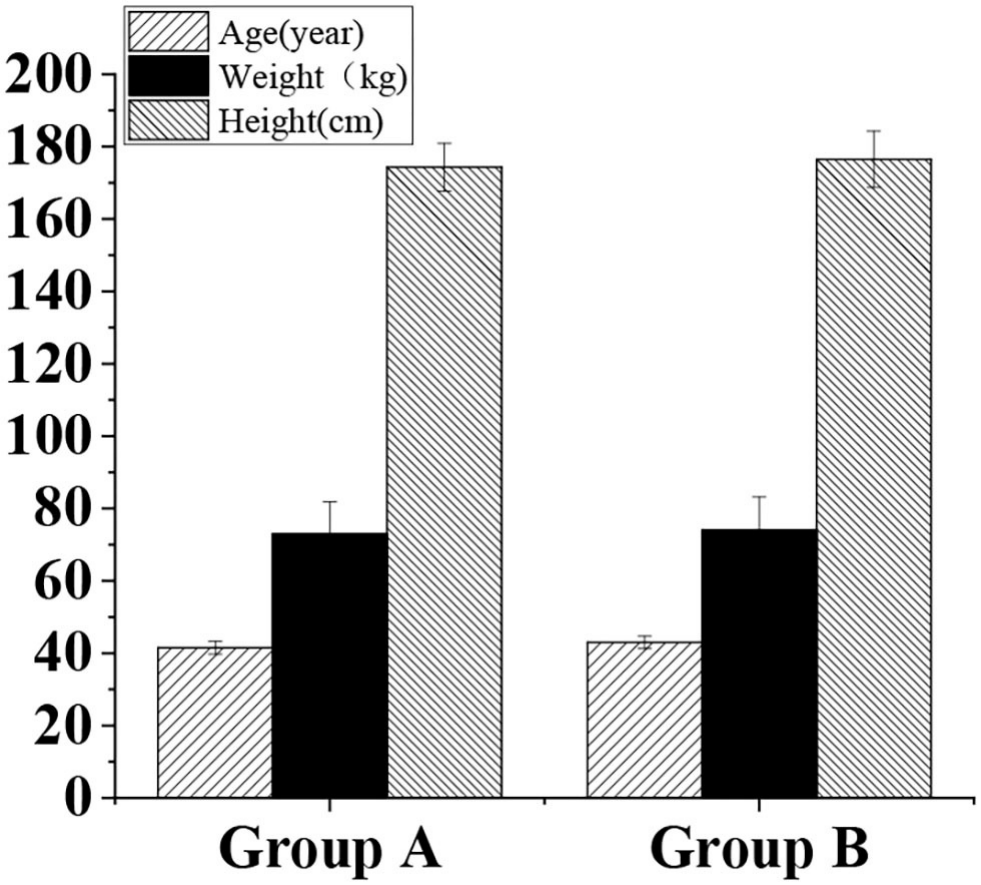
The basic physical information of included subjects.

In total, 100 cases in the age group of 20–45 years of units A and B were considered the experimental subjects. The basic information is shown in Figure 1. The age (40–45 years) and units (unit A and unit B) of the test subjects were selected randomly. It can explain the feasibility of the evaluation model and avoid the tendency to be confused when there are too many subjects. For different age groups, the same methods and different criteria for the evaluation were employed. Therefore, any age group can select the subjects of two representative units for the evaluation, so it is easy to for comparison between units.

### Data Source

The physical fitness test is the most important means to understand the physical health of the occupants. The national physical fitness benchmarks are selected in this study to determine the appropriate physical fitness index system, including the following indicators: blood pressure and heart rate (to evaluate the function of the vascular system for measuring blood pressure and heart rate, reflecting the quality of the residents' heart), grip strength (to evaluate upper limb muscle strength), reflected value (to evaluate the response speed), sitting posture (to assess the state of abdominal muscles), vital capacity (to measure the vital capacity using electronic speedometer), maximum oxygen uptake (to evaluate cardiovascular endurance and aerobic capacity), and bone density (to evaluate the bone density).

At present, the incidence of cardiovascular and metabolic chronic diseases is very high. Therefore, the health diagnosis system to implement the questionnaire survey of chronic diseases includes various chronic diseases (as shown in Table 1).

**Table 1:** Chronic disease survey scale of resident.

**Serial no**.	**Item**		
Q 1	Hypertension	Yes	No
Q 2	Arteriosclerosis	Yes	No
Q 3	Osteoporosis	Yes	No
Q 4	Diabetes	Yes	No
Q 5	Fatty liver	Yes	No
Q 6	Coronary heart disease	Yes	No
Q 7	Hyperlipidemia	Yes	No
Q 8	Gout	Yes	No

In the system, the result is evaluated with “Yes” or “No,” so the length of the result is 1. Before the questionnaire, “No” is determined by default. After the user selects the corresponding chronic disease, the result of the disease is displayed as “Yes.”

### Construction Model of Intelligent Exercise Prescription System

**Figure 2:**
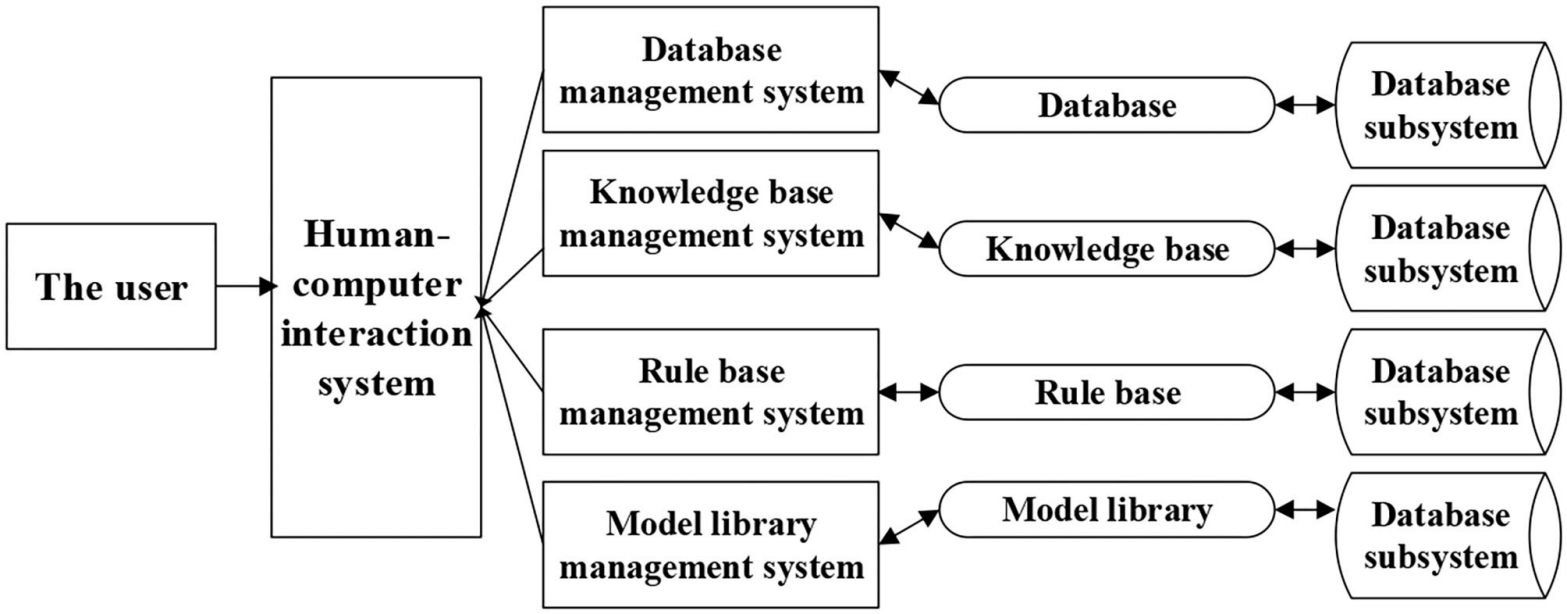
The overall configuration of the smart exercise prescription system.

The configuration of the DSS refers to the method of combining the components of the DSS with each other. There are various DSS frameworks based on the actual demands, mainly including the subsystem-based framework and knowledge-based framework. The structure constitutes to the system with its own strengths and weaknesses, and determines what kind of system structure to use. The objective of this study was to develop a decision support that aims to provide users with a method of improving physical health. Based on the characteristics of the operation process and the knowledge (data), the structure based on the subsystem library is adopted to design, construct, and implement the system. The whole system is composed of three layers, namely, resource layer, information processing layer, and human–computer interaction (HCI) layer. Figure 2 shows the overall configuration of the smart exercise prescription system.

As shown in Figure 2, in the hierarchical structure, the resource base is mainly composed of database, knowledge base, rule base, and model base. Resource management consists of 4 library management systems, and each management system is associated with the correct, reasonable, and convenient call of resources. The HCI layer is a convenient entrance to realize the direct dialogue between the system and the user and provide the user's decision support. Exercise prescription is personalized, so the data required are mainly composed of personal-related basic information, physical fitness evaluation data structure, and chronic disease data structure evaluation data. The basic personal information includes age, gender, occupation, and other information, as shown in Table 2.

**Table 2:** Personal information.

**Field name**	**Type**	**Length**	**Remark**
ID-code	varchar	16	NotNull
Name	varchar	18	NotNull
Gender	varchar	2	NotNull
Age	datetime	30	Null
Marital status	varchar	60	Null
Profession	varchar	60	Null
Education	varchar	60	Null

As presented in Tables 3, 4, there are 5 columns to show the data structure. The first column contains the field names; the second column contains the field attribute definitions; the third column is a simple explanation of the corresponding field of the “command” (sometimes, the foreign language is translated into the local language); the fourth column is “type,” which refers to the character data type of the field; and the data type of the system mainly adopts the Varchar data type, which is characterized by variable text within the specified character length (column 5).

**Table 3:** Structure on fitness assessment data.

**Field name**	**Item**	**Type**	**Length**	**Remark**
Blood pressure	Blood pressure	Varchar	4	Null
Grip strength	Grip strength	Varchar	3	Null
The whole body reaction	Systemic reaction	Varchar	3	Null
sit-ups	Sit-ups	Varchar	5	Null
Lung capacity	Vital capacity	Varchar	3	Null
Maximum oxygen uptake	Maximal oxygen uptake	Varchar	5	Null
Bone mineral density	Bone density	Varchar	3	Null

**Table 4:** Structure on chronic disease data.

**Field name**	**Item**	**Type**	**Length**	**Remark**
Hypertension	Hypertension	varchar	1	Null
Hardening of the arteries	Arteriosclerosis	varchar	1	Null
Osteoporosis	Osteoporosis	varchar	1	Null
Diabetes	Diabetes	varchar	1	Null
Fatty liver disease	Fatty liver	varchar	1	Null
Coronary heart disease	Coronary heart disease	varchar	1	Null
Hyperlipidemia	Hyperlipidemia	varchar	1	Null
Gout	Gout	varchar	1	Null

### Statistical Analysis of Data

The SPSS is adopted to make statistics on residents' fitness test data. The Microsoft Excel 2003 software is applied to solve the matrix operation in the analytic hierarchy process and fuzzy comprehensive evaluation method. The Ridit method is used to test the significance of the grade judgment result.

### Data Model

**Figure 3:**
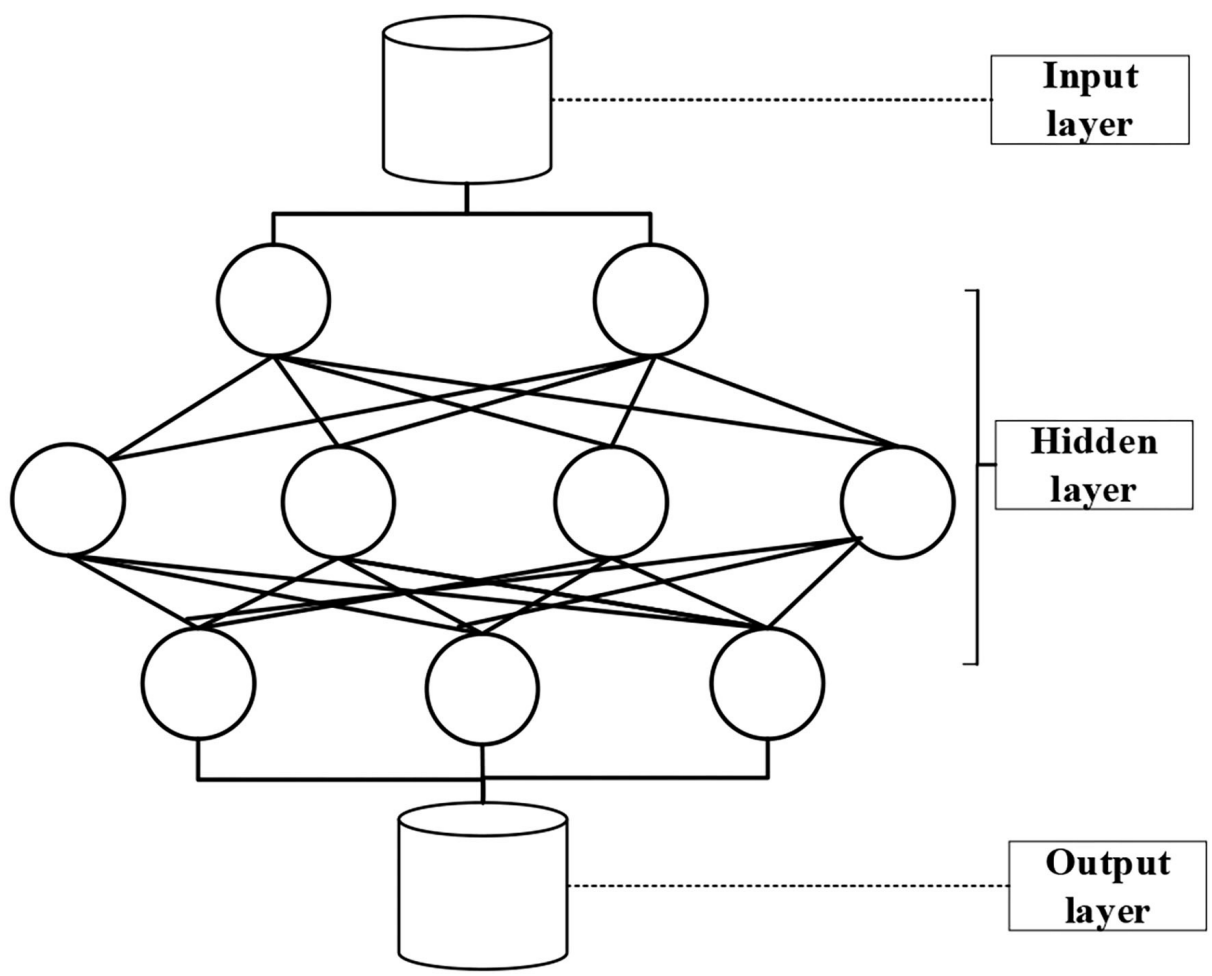
The basic structure of a three-layered BPNN.

Currently, the commonly used data mining methods mainly include artificial neural network (ANN) and association rule data models. The ANN is a theoretical mathematical model of the human brain and its activities. It consists of a large number of processing units interconnected in an appropriate manner, and it is a large-scale nonlinear adaptive system. BPNN algorithm is widely used in many fields such as medical treatment and industry because of its large-scale parallel processing, good self-organization, and self-learning ability and flexibility. BPNN algorithm is the main idea of the learning algorithm. After the learning samples are inputted, the attribute algorithm adjusts the network weight and training deviation to be as close as possible to the vector output vector and the expected training value. The output layer of the square error network is less than the specified error. Then, the deviation of the network weight is saved. The traditional BPNN algorithm can perform one-dimensional mapping in a three-layered network. However, it contains inherent defects such as not fast enough and errors, so that it is easy to fall into the local network with the smallest result. Therefore, the practicality of the BPNN is basically an improved version of the algorithm. Both the improved BPNN algorithm and the traditional BPNN algorithm have the same basic network structure (as illustrated in Figure 3).

Due to some shortcomings of the traditional BPNN, it is optimized using the Bamberg algorithm. The Venberg–Marquart algorithm replaces the mean square error (MSE) of the sum of square error (SE) and least square error (LSE). The variance is expressed as follows:


(1) $$\begin{array}{l} E=\underset{C}{\sum }\left(\gamma^{\rho }\right)=\frac{1}{2}\iint \delta \end{array}$$

where *E* is the sum of squared errors, and γ refers to the vector of element ^ρ^;

It is assumed that the current position spreads and moves to the new position μ ^*N*+1^, if the amount of movement μ *N-1* is small, then β can be expanded into a first-order Tailor series, as follows:


(2) $$\begin{array}{l} \beta \left(\mu^{N+1}\right)=\left(\mu^{N}\right)+\eta \left(\mu^{N+1}-\mu^{N}\right) \end{array}$$

where *N*+*1* represents the current weight value or threshold.


(3) $$\begin{array}{l} {\left(J\right)}_{DI}=\frac{\chi \beta^{\eta }}{\chi \eta^{\eta }} \end{array}$$

where χη^η^ represents the weight or threshold of the first element. Therefore, the error function is as follows:


(4) $$\begin{array}{l} E=\frac{1}{2}{\left||\chi \left(\mu^{n}\right)+j\left(\mu^{N+1}-\mu^{N}\right)|\right|}^{2}+\lambda ||\mu^{N+1}-\mu^{N}|| \end{array}$$

where χη^η^ is a normal number, and the following equation can be obtained by finding the minimum point of N+1:


(5) $$\begin{array}{l} \mu^{N+1}=\mu^{N}-{\left(J^{\gamma }=JI\right)}^{-1}J^{\gamma }\mu \delta \left(\mu^{n}\right) \end{array}$$

where *I* refers to the identity matrix. It should be adjusted for each iteration of the algorithm. To ensure that (*J*^γ^ = *JI*)^−1^ is reversible and positive, *J* has to be large enough. The larger the *J* value, the more the algorithm adjusts the weights similar to the gradient descent method.

### Generative Model of Health Guidance Model

According to the influence mechanism of many factors on physical fitness, the following neural network model can be obtained as follows:


(6) $$\begin{array}{l} D_{G}=(\text{A},\text{S},\text{SU},\text{LC},\text{BMD}) \end{array}$$

where A is the age, S is the gender, SU refers to the sit-up score, LC refers to the vital capacity value, and BMD represents the bone density value. This is a simple model organically generated based on health guidance under ANN. To prevent the occurrence of different unit dimensions or the phenomenon of neuron saturation, the various factors in the model are standardized as follows:


(7) $$\begin{array}{l} z_{i}=\frac{\gamma_{i}-\gamma_{mix}}{\gamma_{\max}-\gamma_{\min}}\alpha +\beta \end{array}$$

where, the variables before and after the conversion are *z*_*i*_ and γ_*i*_, respectively; the maximum and minimum values of γ_*i*_ are respectively γ_max_ and γ_min_, respectively; and α is a parameter between 0 and 1, so the input value *z*_*i*_ is obtained in the interval (0,1).

### Data Display

Under different data requirements, the data entering the database have to create a corresponding transformation in the conjugate difference product format. According to the needs of the operation, this system has data in three forms.

The first form refers to original presentation of the data. The display of raw data is mainly physical index test data and medical index input information. There are three types of display of raw data, namely, field name (index name), data volume, and numerical unit. The chronic questionnaire data are shown as 0 and 1.

The second form refers to the hierarchical display of data. The level of data refers to the classification of the original data into different levels according to a certain rule. According to different genders and ages, the physical health standards can be graded into five levels (i.e., A, B, C, D, and E): excellent, good, medium, poor, and very poor. For medical indicators, A, B, and C are normal, low, and high for residents of different ages. The survey results of chronic diseases are not used as the basis of exercise prescription, so there is no corresponding grade and score.

The third form refers to the score display of the data. To evaluate the overall physical state of the user, it is necessary to quantitatively measure the raw results of the indicators and evaluate the overall physical state based on the data. This is a composite score. In the study of national physique, there are many people who directly quantify the results of grade evaluation. Five levels (i.e., excellent, good, medium, poor, and very poor) are assigned with 5, 4, 3, 2, and 1 points, respectively. The higher the score, the better the physical condition indicated by this indicator.

If a prescription is required, a physical test has to be performed, and then the system interface is inputted to read the result of the physical test. At the same time, the life questionnaire survey is performed. After the physical examination and questionnaire are over, the personal information is entered into the database. The knowledge of the database is consistent with the contents of the knowledge base, forming personal physical information. The personal composition information is consistent with the indicators based on the evaluation system. The composition state is inferred through the inference machine, and various methods are evaluated. The system that obtains each physical state selects an appropriate exercise prescription model according to the health state of each individual. In the case of multiple exercise prescription intervention items, the most suitable system is selected through the system dynamics model manual, and the feedback is given to the user to guide the advancement of the patient's health goals and exercise prescription.

### Statistical Analysis

The SPSS19.0 and AMOS software are adopted for statistical analysis. The χ2 test is used to compare the differences among the survey results of the doctor–patient relationship. Corrected item-total correlation (CITC) value, Cronbach's alpha test consistency, and exploratory factor are applied to test the reliability and validity of the designed questionnaire. Then, the structural equation model in the analysis of moment structure (AMOS) software is adopted to verify the influencing factor model constructed in this study. When *P* < 0.05, the difference is considered statistically significant.

## Results

### Physical Fitness Test Results of Residents

**Figure 4:**
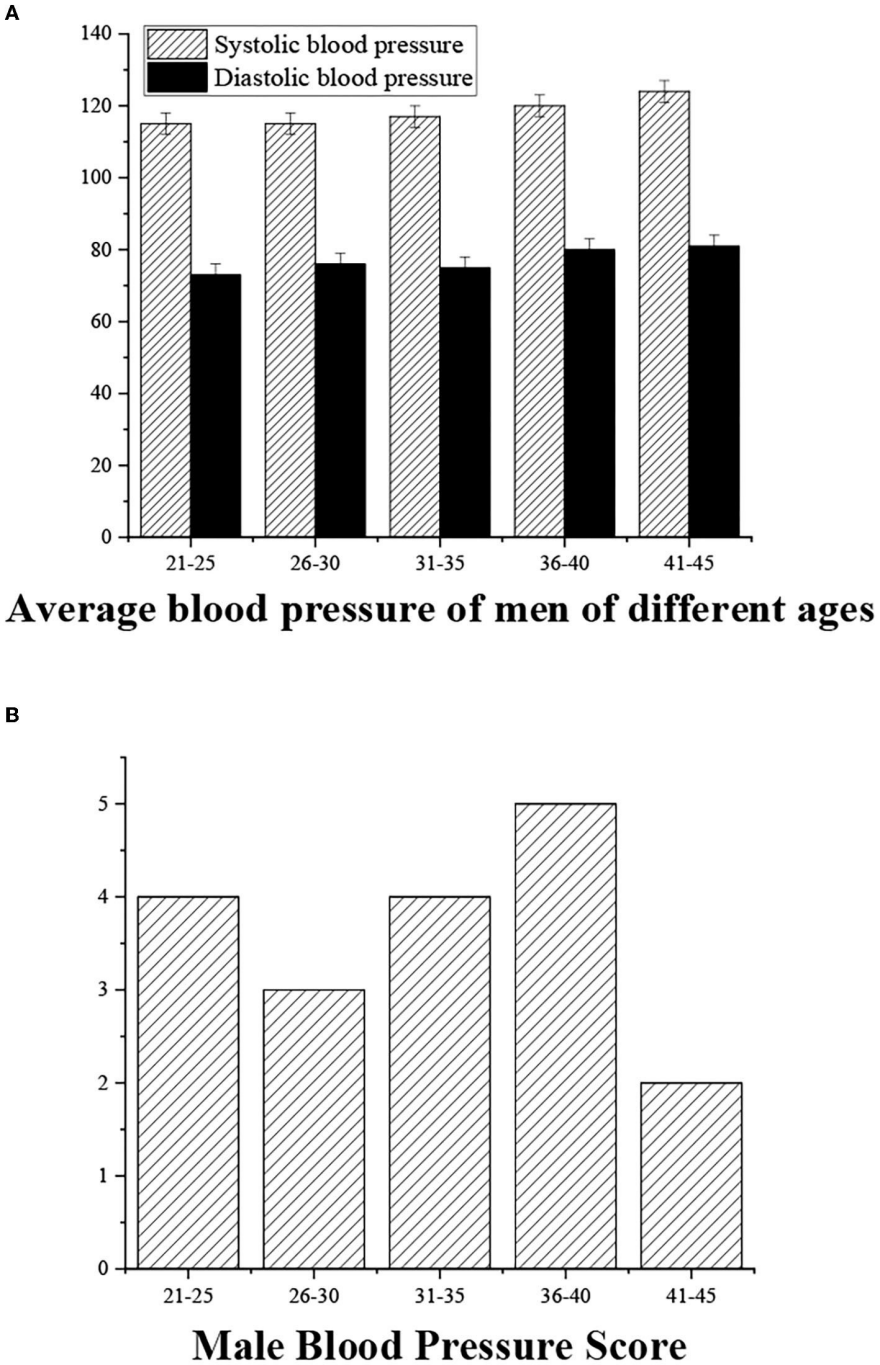
**(A)** Statistical average blood pressure (SBP/DBP) for men of different ages. **(B)** Blood pressure measurement score.

**Figure 5:**
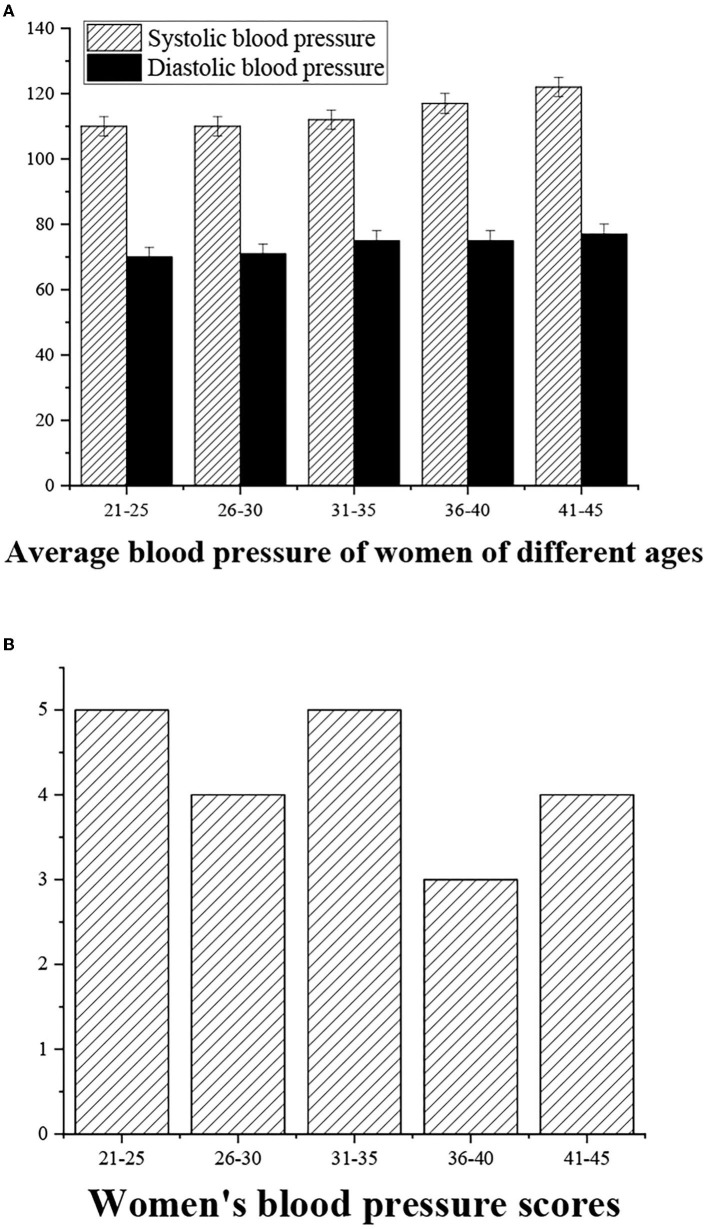
**(A)** Statistical average blood pressure (SBP/DBP) for women of different ages. **(B)** Blood pressure measurement score.

The Chinese guidelines for the prevention and treatment of hypertension provide a sufficient scientific basis for setting the population with systolic blood pressure (SBP) of 120–129 mmHg and diastolic blood pressure (DBP) of 80–84 mmHg to the range of high normal values. In view of this, the classification of blood pressure is as follows. SBP < 120 is determined as “excellent” (5 points), SBP = 120–130 means the health status is “good” (4 points), SBP = 130–140 or <90 means the health status is “medium” (3 points), SBP > 140 is determined as “alert” (2 points); DBP < 80 is defined as “excellent” (5 points), DBP = 80–85 means the blood pressure is “good” (4 points), DBP = 85–90 or <60 is blood pressure is “medium” (3 points), and DBP > 90 means it is “alert” (2 points) (Figures 4, 5).

**Figure 6:**
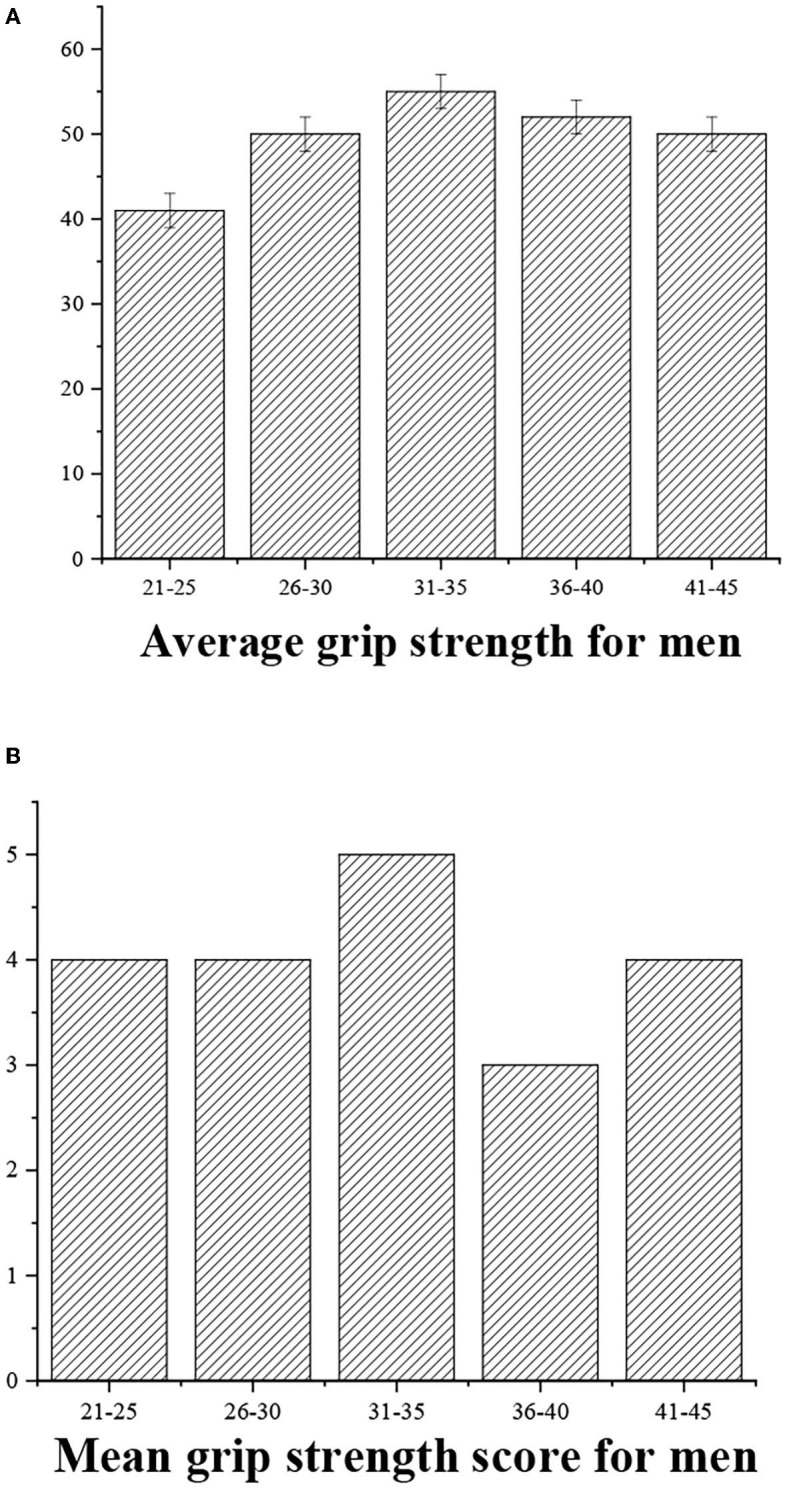
**(A)** Statistical average grip strength for men of different ages. **(B)** Grip strength score.

**Figure 7:**
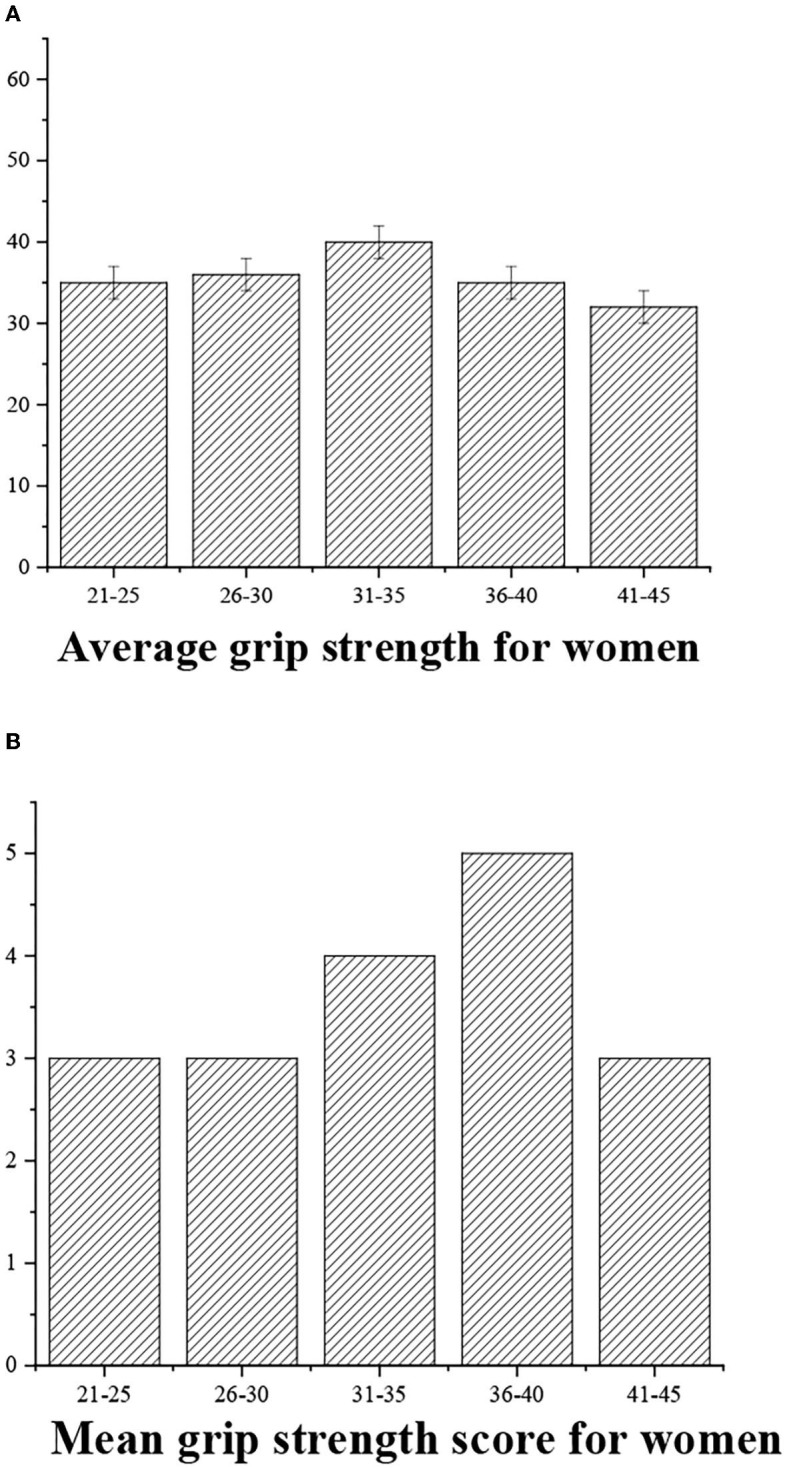
**(A)** Statistical average grip strength for women of different ages. **(B)** Grip strength score.

The grip strength can be graded as follows: >55 (“excellent,” 5 points), 40–50 (“good,” 4 points), 30–40 (“medium,” 3 points), <30 (“general,” 3 points), and <20 (“alert,” 2 points) (Figures 6, 7).

**Figure 8:**
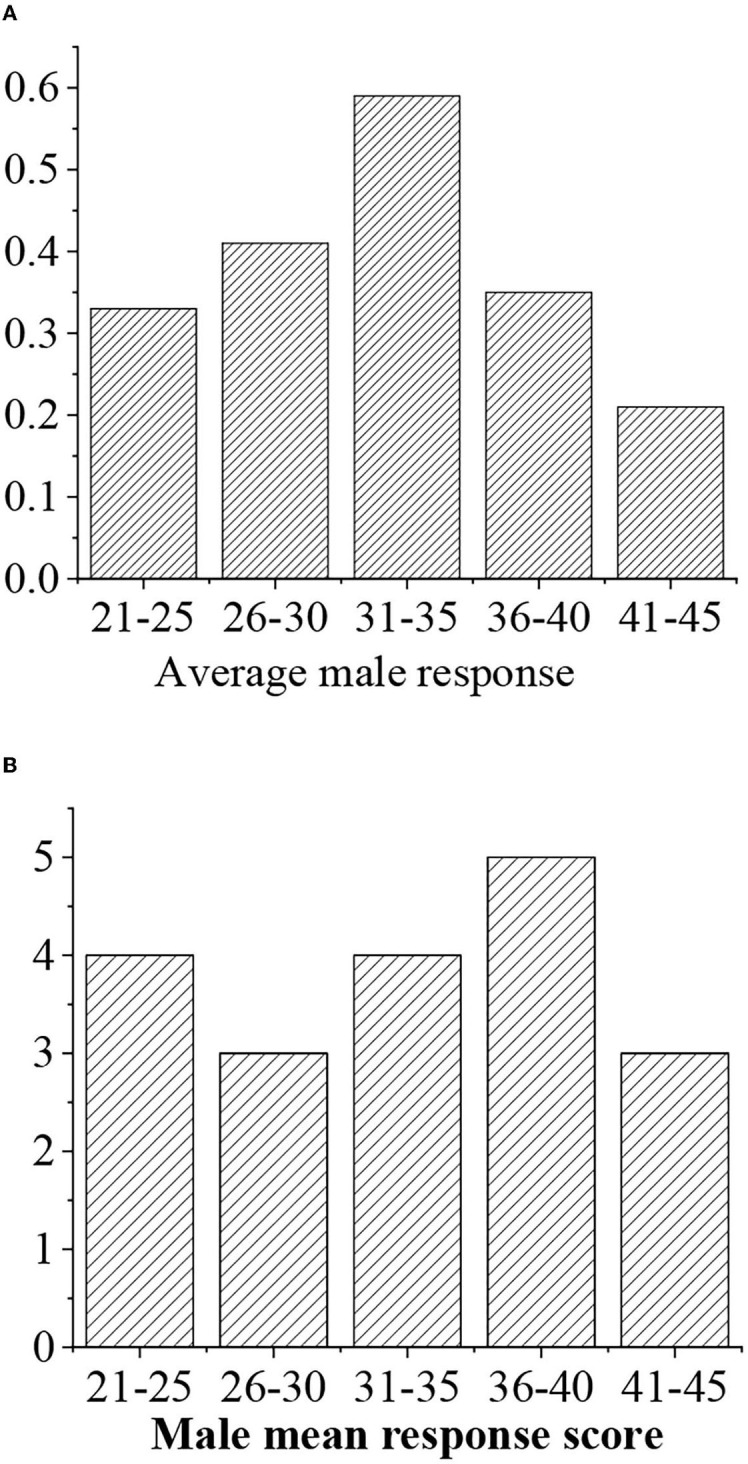
**(A)** Statistical average response value for men of different ages. **(B)** Response value score.

**Figure 9:**
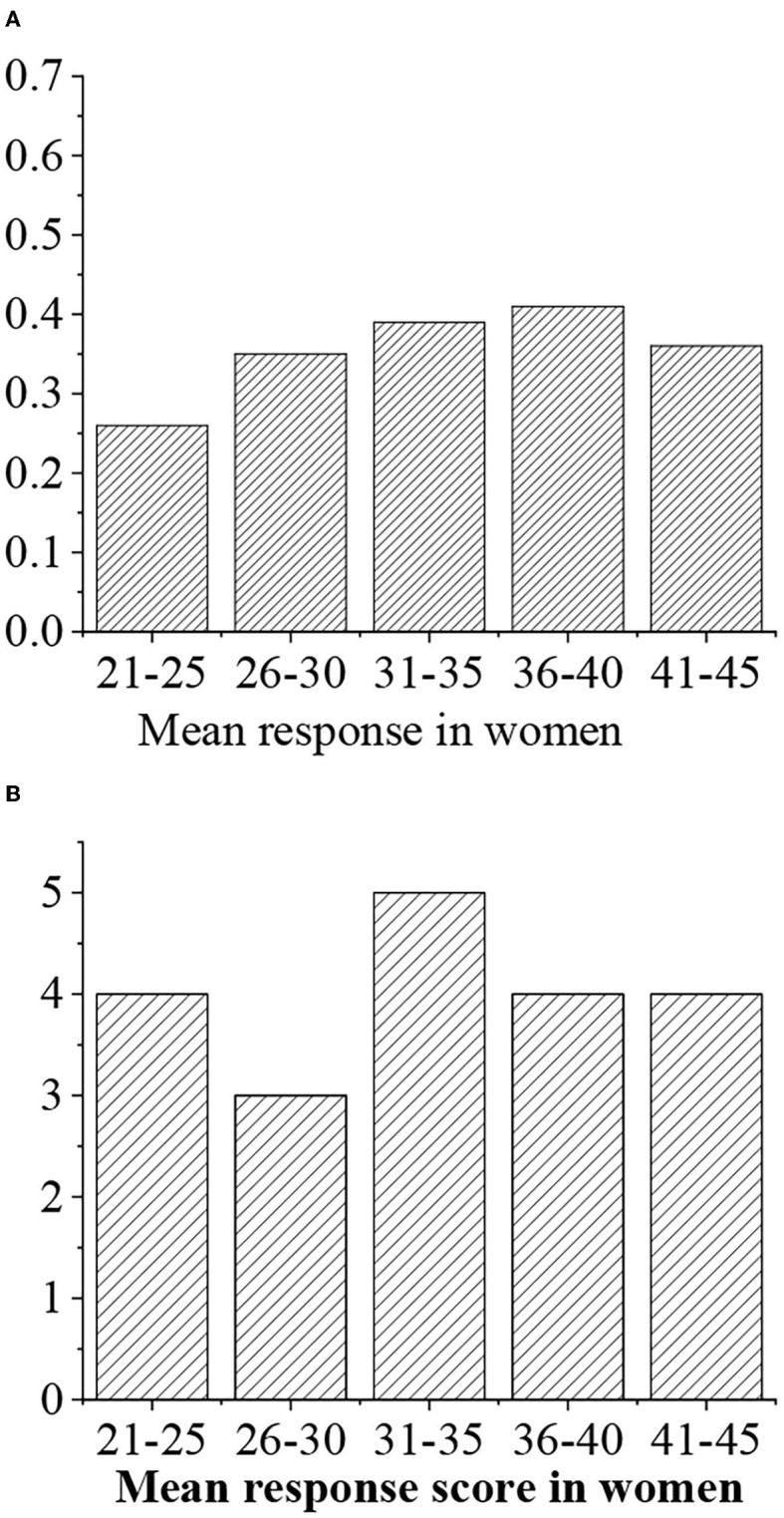
**(A)** Statistical average response value for women of different ages. **(B)** Response value score.

The average response value is divided into four levels, namely, >0.7 (“excellent,” 5 points), 0.6–0.7 (“good,” 4 points), 0.5–0.6 (“medium,” 3 points), <0.5 (“general,” 3 points), and <0.4 (“alert,” 2 points) (Figures 8, 9).

**Figure 10:**
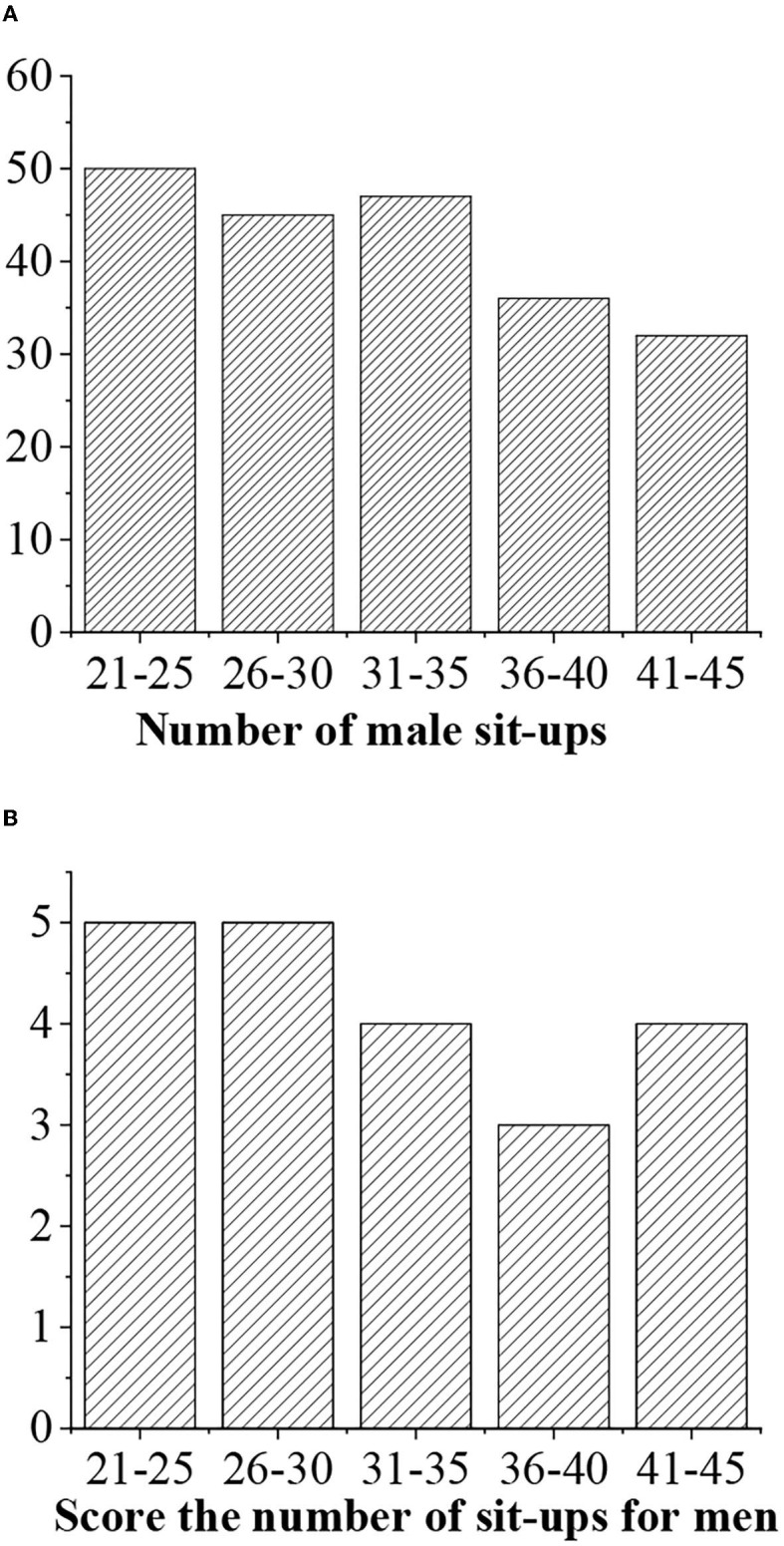
**(A)** Statistical average sit-ups value for men of different ages. **(B)** Sit-ups score.

**Figure 11:**
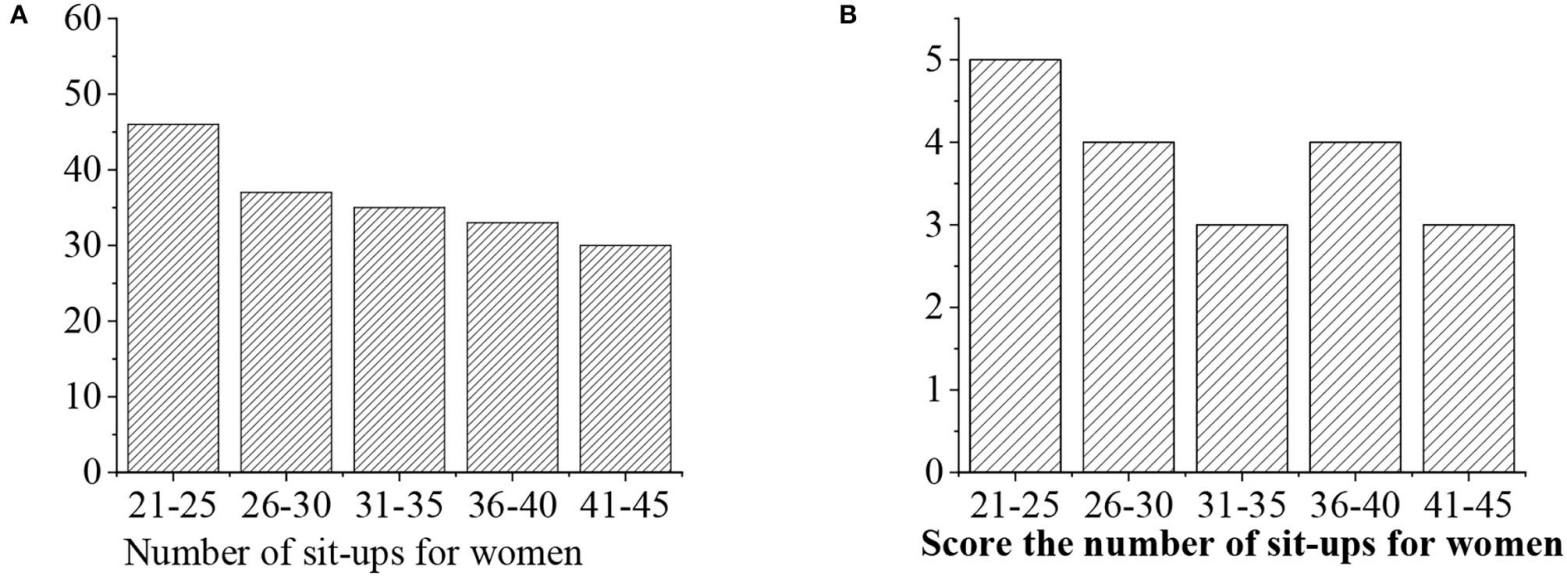
**(A)** Statistical average sit-ups value for women of different ages. **(B)** Sit-ups score.

The grades of sit-ups are given as follows: >50 (“excellent” with 5 points), 40–50 (“good,” 4 points), 30–40 (“medium,” 3 points), <40 (“medium,” 3 points), and <30 (“alert,” 2 points) (Figures 10, 11).

**Figure 12:**
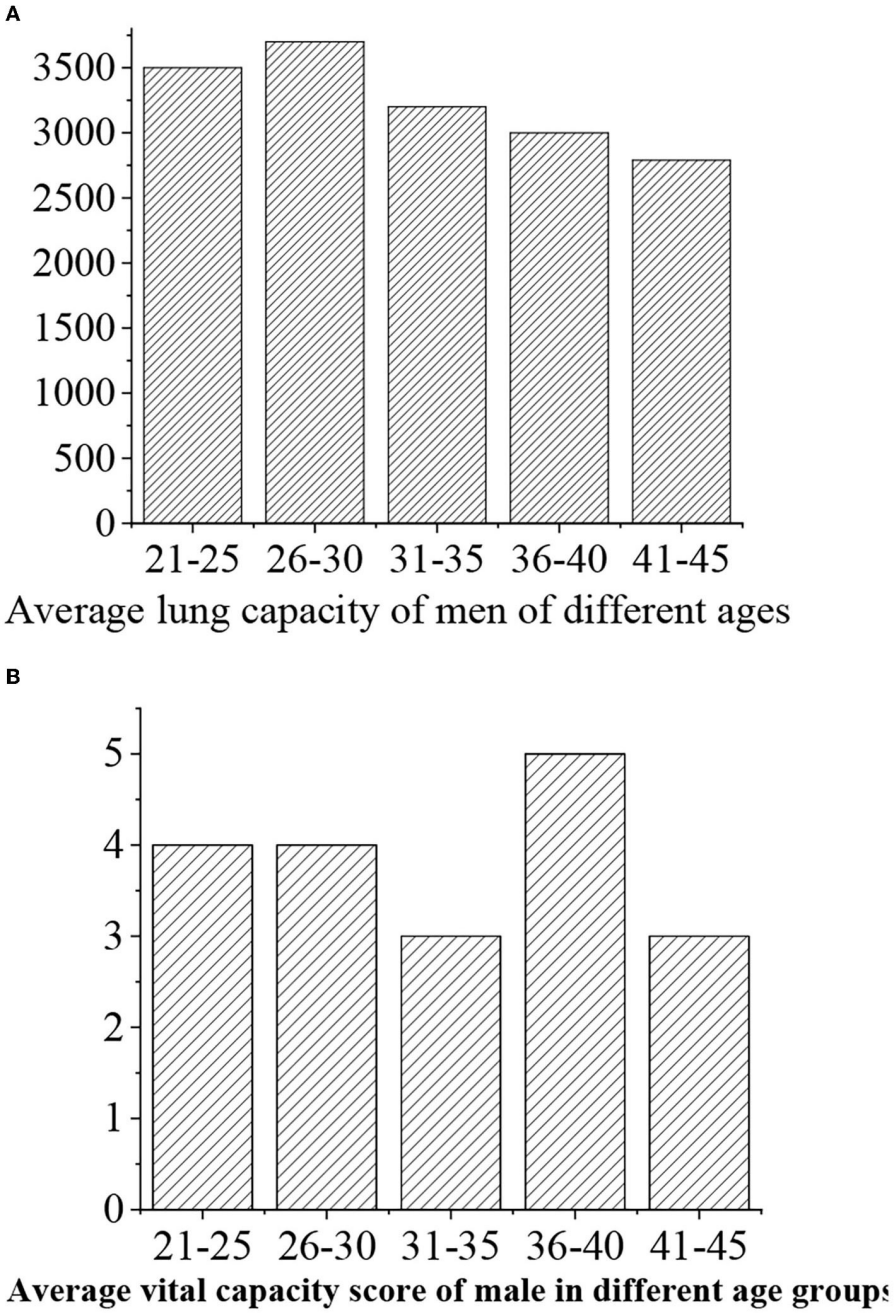
**(A)** Statistical vital capacity for men of different ages. **(B)** Vital capacity score.

**Figure 13:**
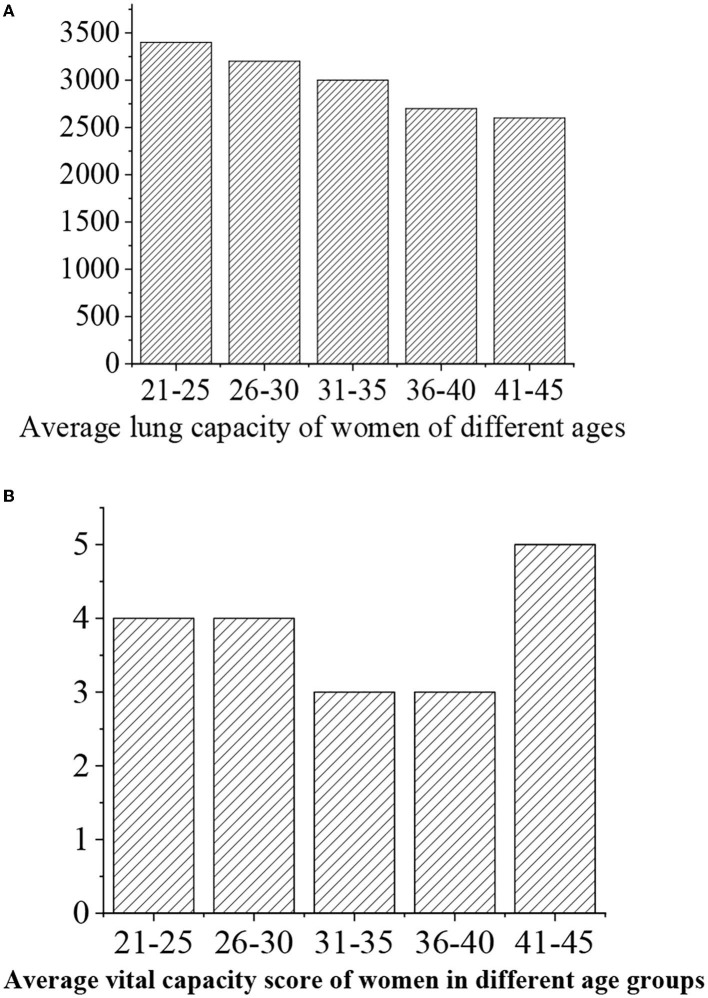
**(A)** Statistical vital capacity for women of different ages. **(B)** Vital capacity score.

The grades of vital capacity are given as follows: >3,500 (“excellent,” 5 points), >3,400 (“good,” 4 points), 3.400–3,300 (“medium,” 3 points), 3,300–3,200 (“general,” 3 points), and <3,200 (“alert,” 2 points) (Figures 12, 13).

**Figure 14:**
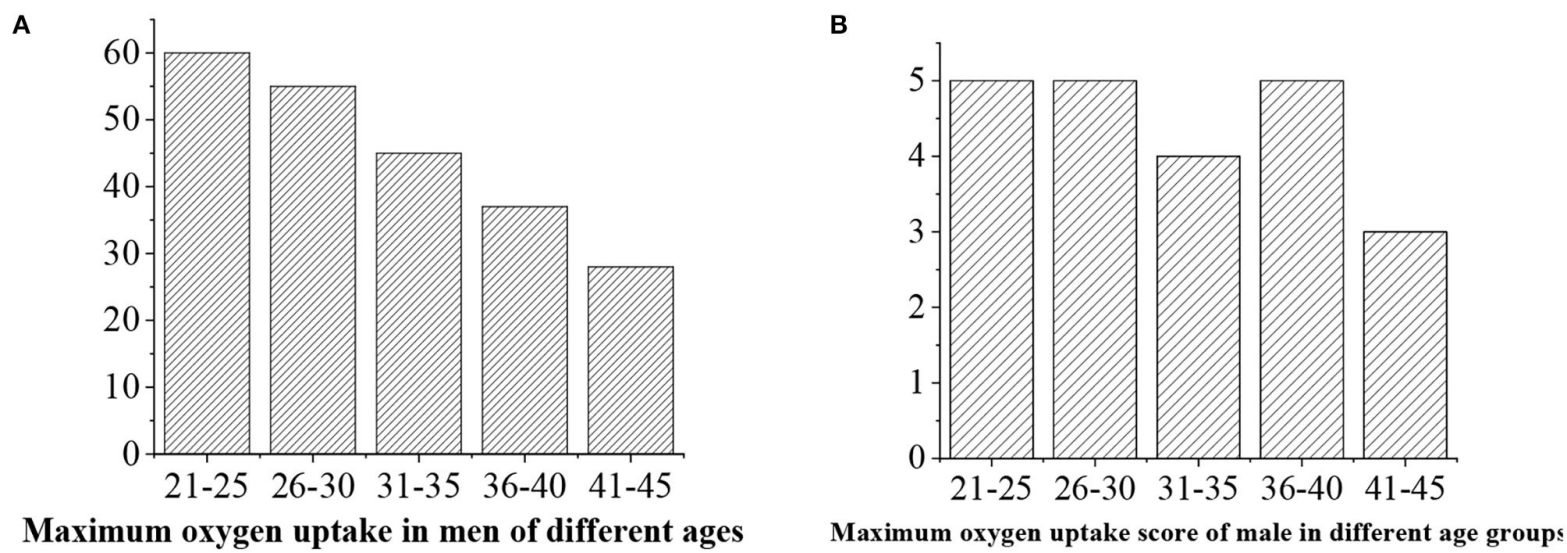
**(A)** Statistical average maximum oxygen uptake value for men of different ages. **(B)** Maximum oxygen uptake score.

**Figure 15:**
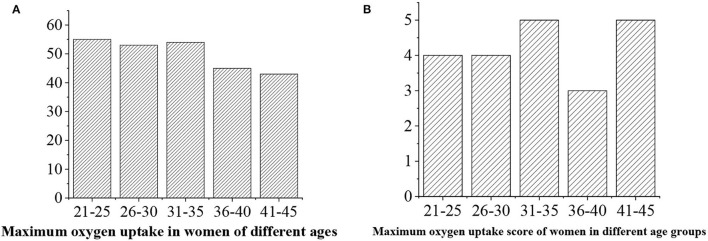
**(A)** Statistical average maximum oxygen uptake value for men of different ages. **(B)** Maximum oxygen uptake score.

The maximum oxygen uptake is divided into five levels, namely, >50 (“excellent,” 5 points), 40–50 (“good,” 4 points), 35–40 (“medium,” 3 points), 30–35 (“general,” 3 points), and <30 (“alert,” 2 points) (Figures 14, 15).

**Figure 16:**
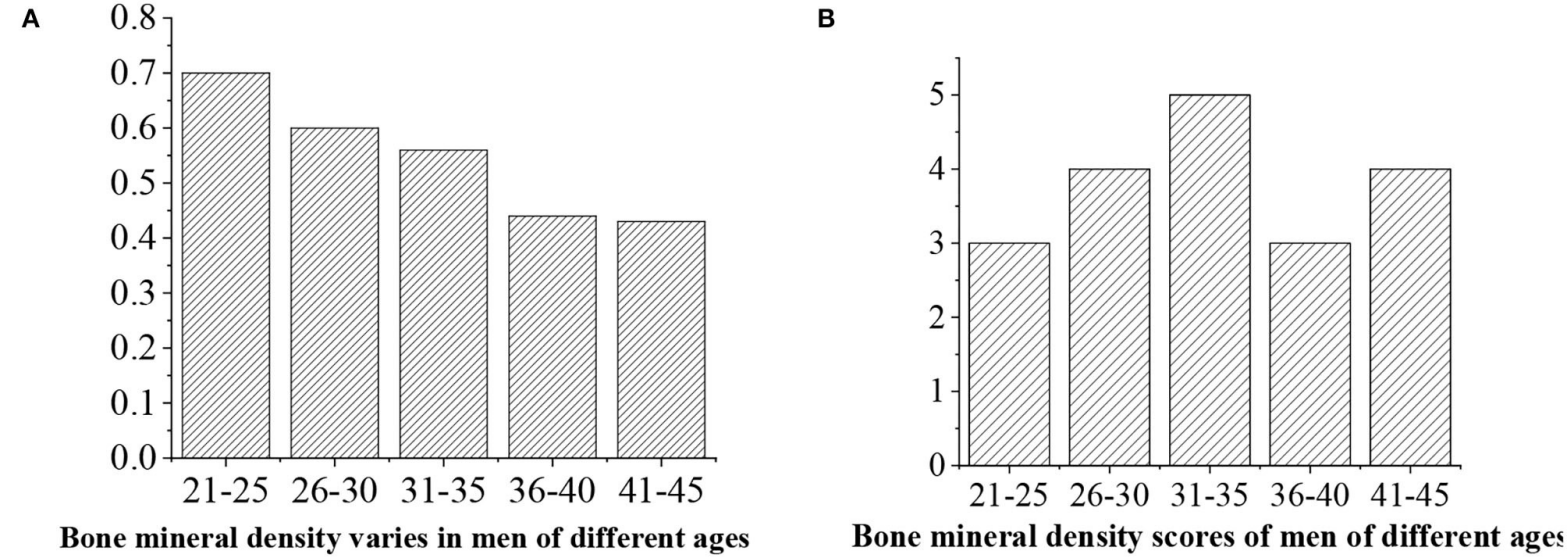
**(A)** Statistical average bone density value for men of different ages. **(B)** Bone density score.

**Figure 17:**
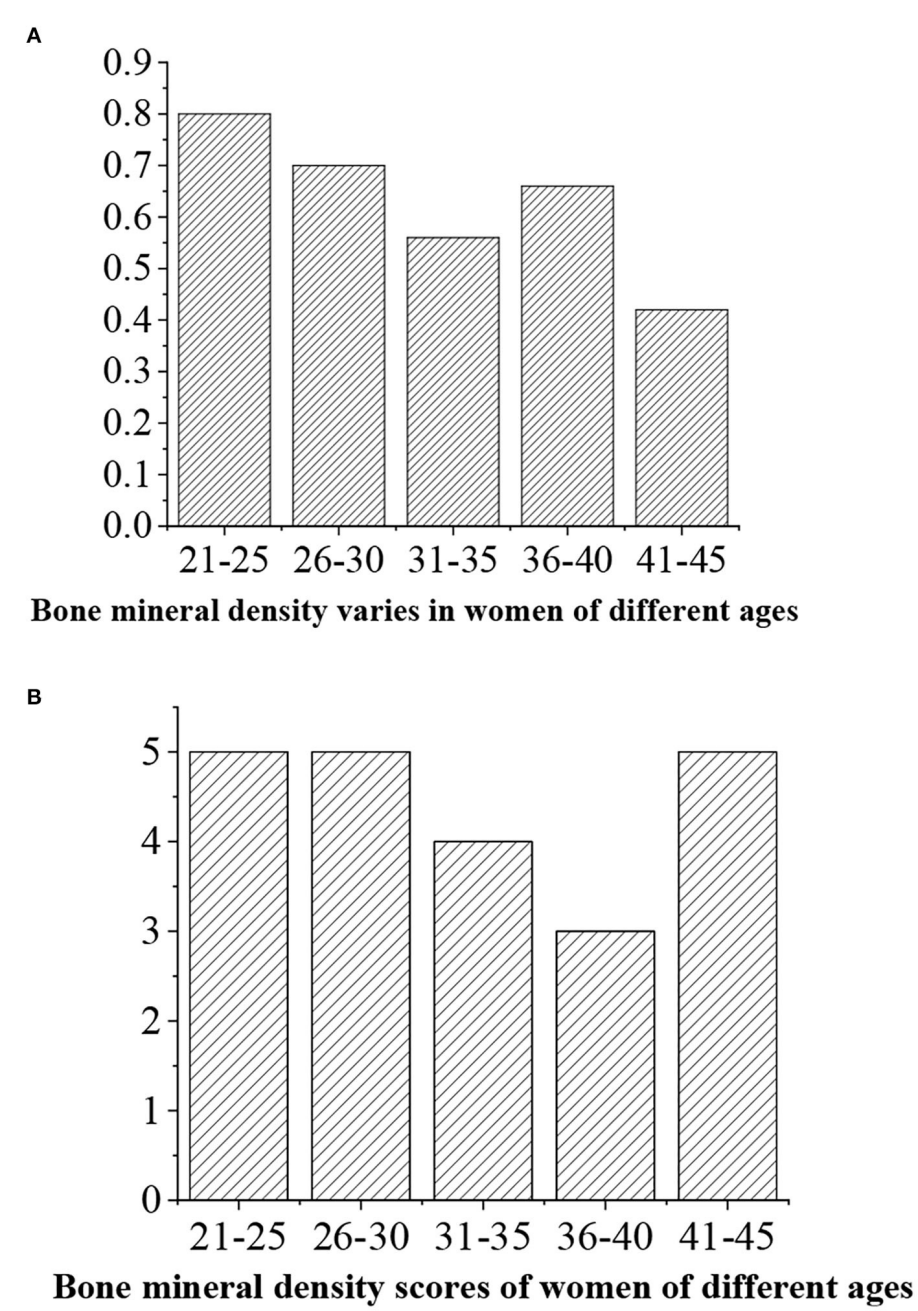
**(A)** Statistical average bone density value for women of different ages. **(B)** Bone density score.

The levels of bone density are given as follows: >0.6 (“excellent,” 5 points), 0.5–0.6 (“good,” 4 points), 0.4–0.5 (“medium,” 3 points), 0.4–0.5 (“general,” 3 points), and <0.4 (“alert,” 2 points) (Figures 16, 17).

### Analysis of the General Situation of Residents' Chronic Diseases

**Figure 18:**
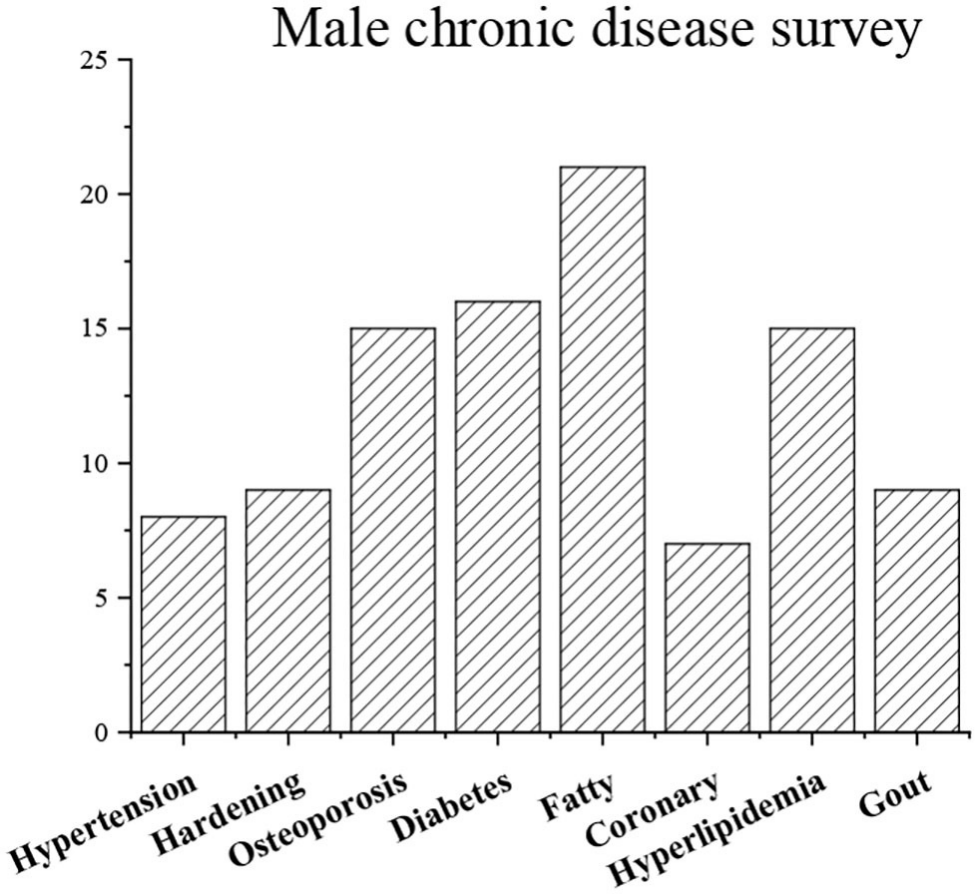
General situation of residents' chronic diseases (man).

**Figure 19:**
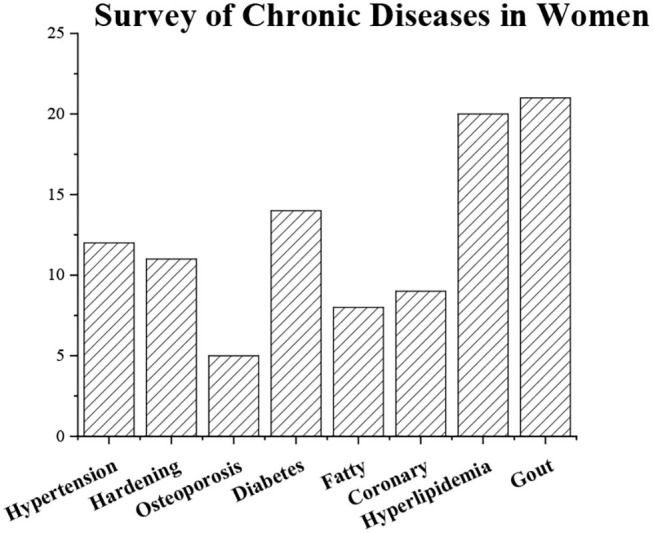
General situation of residents' chronic diseases (woman).

The results of the residents' chronic disease questionnaire are shown in Figures 18, 19. They indicate that the number of patients with obesity is the largest, while the number of patients with coronary heart disease is the smallest (*P* < 0.05), and female patients have the most symptoms of gout (*P* < 0.05).

### Questionnaire Test Results

A test on reliability and validity of the scales designed in this study showed that the CITC value >0.6, Cronbach's alpha >0.7, Kaiser-Meyer-Olkin (KMO) >0.7, and Bartlett sphere test <0.05. It shows that the reliability, validity, and fitting effect of each item in the scale designed in this study can meet the requirements.

### Promotion of Decision Support by Physique Health of the Platform

The idea of providing decision-making is to firstly determine whether the subject suffers from hypertension based on the evaluation results. If the blood pressure is too high (to facilitate the follow-up evaluation, a chronic disease questionnaire survey is performed first on subjects before testing and evaluation, which has become one of the important bases for subsequent formulation of prescriptions), the decision support provided first gives the decision-making based on the degree of hypertension. Moreover, the first thing to be advertised to the testees is that when some people know that they have high blood pressure and give up some sports activities, it will lead to the occurrence of other sports deficiency diseases due to lack of exercise.

### Design and Analysis of the Model

**Figure 20:**
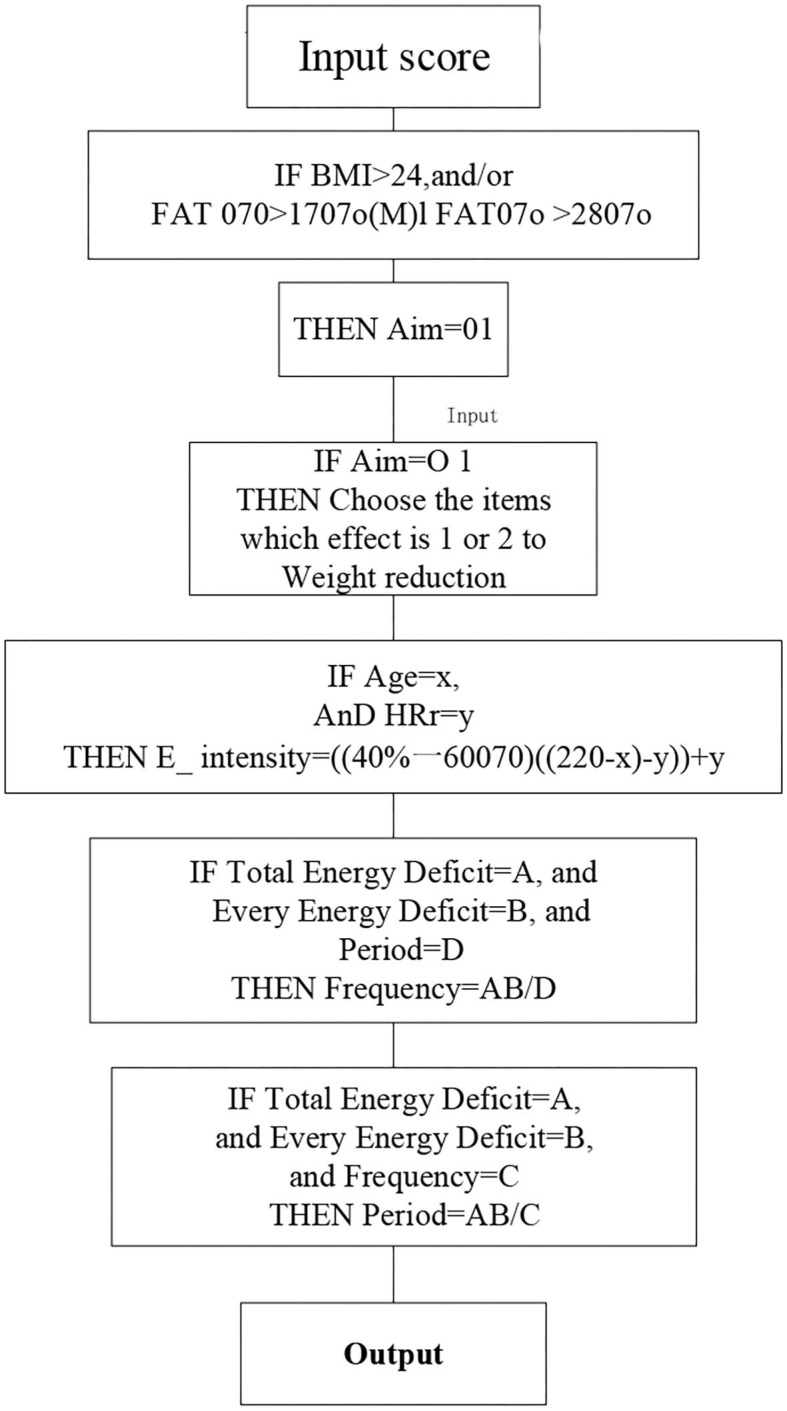
The specific framework of the sport guidance model.

According to the data model generated by AI, the motion guidance framework model is generated as shown in Figure 20. The function of the framework is reflected in the demand for knowledge representation: the model emphasizes the important role and meaning of decision support through input and output, and the framework knowledge does not have such a role. The generated result should include: exercise purpose, exercise item, exercise intensity, exercise time, exercise frequency, exercise period, and exercise precautions. The specific framework of the sport guidance model is given in Figure 20 below.

(1) The exercise prescription model outputs the results based on the 1 or 2 indicators of the physical examination results. Generally speaking, exercise prescription is only a result.
(2) The model is 012, and the sequence of numbers is also the priority level of exercise prescription. The lower the serial number, the higher the priority of its use.

### Evaluation of Physical Function

After the body data are inputted into the platform, the platform will evaluate physical fitness using the AI algorithms. The evaluation of physical function mainly includes 4 items, namely, vital capacity, blood pressure, heart rate at rest, and maximum oxygen uptake, which reflect the status of human cardiopulmonary function. Cardiopulmonary function is a comprehensive reflection of human respiratory system, circulatory system, and muscle work utilization ability, which will seriously affect people's work, life, and even the quality of life.

The main purpose of the assessment of cardiopulmonary function include three aspects, namely, to determine the response of the cardiovascular and respiratory systems to the quiet state and sub-limit and extreme exercise, to provide a basic basis for formulating exercise prescriptions, especially for scientifically determining exercise intensity, and to be undertaken as a check for coronary heart disease.

### Suggestions for Prescription

In this study, the cardiopulmonary function is selected as an example. The prescription recommendations are given as follows: hypertension patients with SBP >180 mm Hg or DBP >105 mm Hg need to be treated with drugs, but nondrug treatment is recommended for blood pressures of 130–180/90–104 mm Hg. Nondrug treatment mainly includes exercise and diet control. Diet should reduce salt intake. In terms of exercise, it is recommended that patients with hypertension do some low-to-medium intensity (40–70%) endurance exercises with a longer duration to consume a lot of calories. When the exercise intensity is determined, the system will ask whether the subject has taken a β-adrenergic receptor resistance agent. If the result of the questionnaire shows that it has been taken, the commonly used (220 - age) cannot be used to calculate the maximum heart rate because such drugs can reduce the maximum heart rate. Instead, an exercise load of 10–12 in the exercise classification test should be used to determine the exercise intensity of 40–60%. Therefore, the decision support of this module is mainly to promote and improve the human heart and lung function.

### Generation of the Evaluation Results

**Figure 21:**
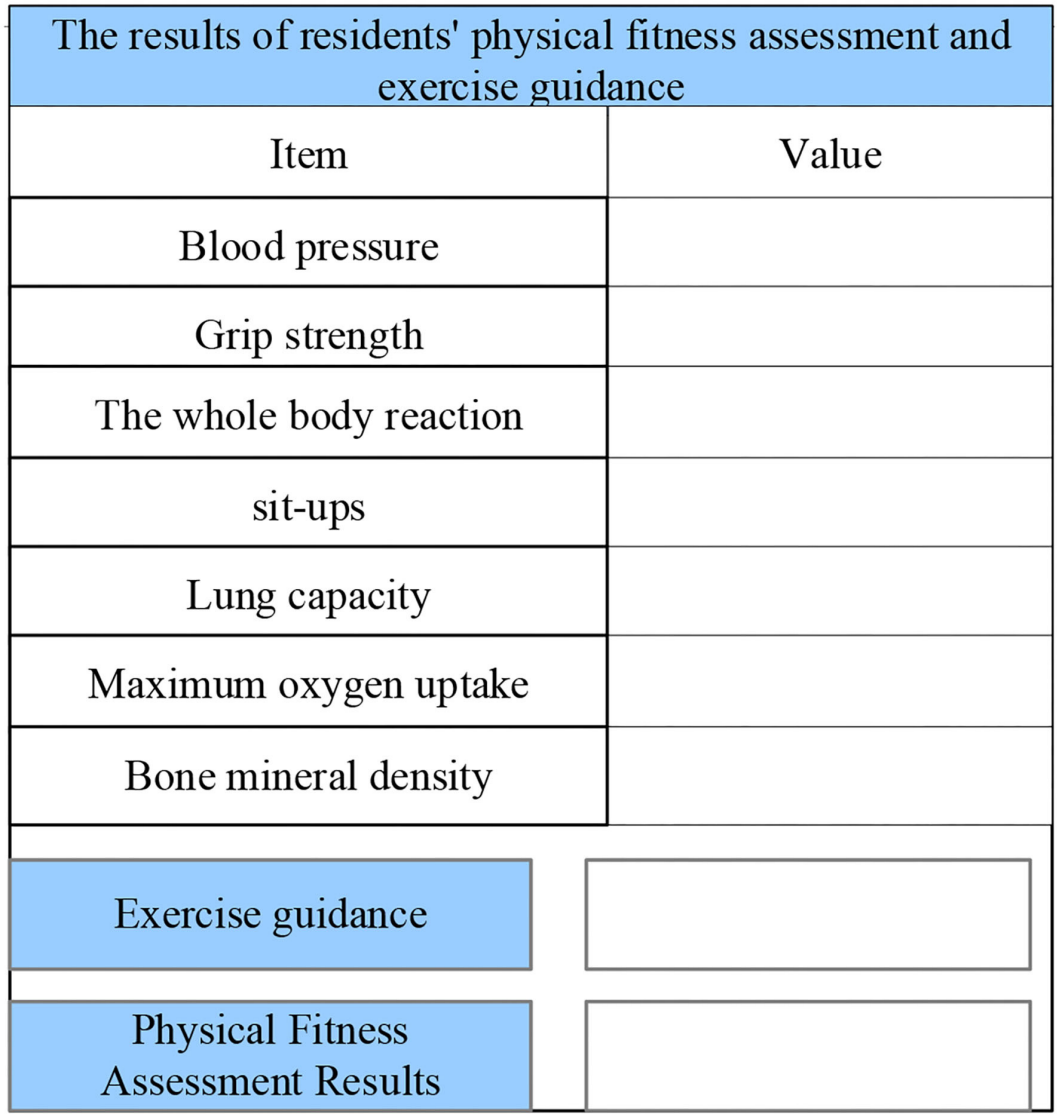
The interface for the generated physical fitness evaluation result.

After the physique test data are generated, the user can select the physique evaluation and diagnosis interface to understand their physique and health status. Figure 21 shows the interface for the generated physical fitness evaluation result. A user can clarify his/her physical by comparison, and it can provide clear goals for users to improve their physical fitness.

## Discussion

The 8,443 physical test results published by the Henan Medical Examination Center reflected the overall situation of the current residents (20–23). The most significant problem is the high proportion of overweight people (accounting for 40.4% of the total number of people), fatty liver (24.3%), and hyperlipidemia (20.4%). The largest proportion of onset age is 40–50 years. The highest incidence of female gynecological diseases is cervicitis (23.0%) and breast hypertrophy (20.6) in 30–50 years old. Psychological investigations show that mildly abnormal interpersonal relationships (60.7%), restlessness (38.6%), depression (35.6%), mild abnormal menopausal symptoms (33.2%), and the proportion of women suffering from the disease are higher than that of men (24–27). According to a survey published by the National Reform Office, the average life expectancy of intellectuals who bear important responsibilities in China was 58 years in 2009, which was lower than the national average life expectancy. In short, obesity is serious, and the health of Chinese residents is not optimistic. The main manifestations are “hypertension, hyperglycemia, hyperlipidemia, and hypoproteinemia” and obesity symptoms. At present, exercise prescriptions have not been unanimously recognized by experts and scholars, and many positive research results have confirmed that physical strength promotion is a very important link. However, the current physical exercise status of residents is worrying, and its physical exercise effect is often difficult to meet the demanding job requirements. Physical health evaluation is a comprehensive evaluation of the physical health of the human body, including physical, mental, and social evaluations, body shape, physical function, physical quality, psychological defects, health, and social adaptation. A comprehensive evaluation method is often used comprehensively judge the physical health. The physical status is currently tested, and the test index is the performance of function and quality. Recently, the morbidity of mental illness among college students has gradually increased, and suicides sometimes occur. Psychological tests in universities have gradually attracted people's attention (28–30). In this study, it is recommended to use BPNN to analyze and evaluate the physique of residents and to make sports recommendation prescriptions for exercise guidance of residents. The innovations of this article are summarized as follows. First, it researches the exercise prescription as a whole, uses different exercise prescriptions as a model, and adopts the framework knowledge representation to realize the integration of production and framework knowledge representation, realizing the unification of knowledge representation and model library. Second, it incorporates the chronic diseases into consideration factors in exercise prescriptions and the AI system, which improves the feasibility and effectiveness of exercise prescription decision support. Third, it uses the AI decision support methods based on BPNN, which enables the system to have a certain degree of self-adaptability, can discover from a large amount of historical data and past experience vital knowledge useful for decision-making, and improve the degree of intelligence of the system and the scientific nature of exercise prescription decision support. Fourth, it applies the system dynamics modeling to physique research and dynamically shows the effect of exercise prescription intervention under the physique system through the system dynamics modeling of physique, providing real-time data support for civil servants to perform exercise prescription and building exercise confidence. Finally, the optimal exercise prescription plan is determined through the simulation of different intervention target combinations.

The limitations of this article are summarized as follows. From the existing data in this study, the database is still very imperfect, especially the collection of medical data, and the imperfect database affects data mining and system dynamics modeling, so the system database needs to be improved and enriched. At present, physical factors such as body shape, physiological functions, and physical fitness are mainly incorporated into the system, so the follow-up studies include psychological factors and social adaptability into the system for improvement. In addition, the exercise prescription in this study is part of the comprehensive evaluation model of individual physical fitness, but there are many qualitative factors that affect physical fitness in addition to the quantitative decision-making given in this study based on data.

## Conclusion

Today is an era of highly developed information, and intelligence is everywhere. The physique and health of Chinese residents also need to be intelligent in the evaluation system, and the emergence of expert systems provides the possibility for intelligent realization. This study adheres to the concept of health management, uses an expert system structure, adopts an object-oriented design method, and applies the Windows Server 2008 as a development platform. The system is developed by using the browser/server structure, based on the MVC development model, and the data and knowledge base rule information are stored in database SQL Server 2005. The user accesses the service provided by the remote server through the browser, so as to build a knowledge base, fact base, and rule base reasoning machine, realizing the data collection of physical fitness test, physical fitness evaluation, health promotion, and information management.

## Data Availability Statement

The raw data supporting the conclusions of this article will be made available by the authors, without undue reservation.

## Ethics Statement

The studies involving human participants were reviewed and approved by Shanghai Lixin University of Accounting and Finance Ethics Committee. The patients/participants provided their written informed consent to participate in this study. Written informed consent was obtained from the individual(s) for the publication of any potentially identifiable images or data included in this article.

## Author Contributions

All authors listed have made a substantial, direct, and intellectual contribution to the work and approved it for publication.

## Conflict of Interest

The authors declare that the research was conducted in the absence of any commercial or financial relationships that could be construed as a potential conflict of interest.

## Publisher's Note

All claims expressed in this article are solely those of the authors and do not necessarily represent those of their affiliated organizations, or those of the publisher, the editors and the reviewers. Any product that may be evaluated in this article, or claim that may be made by its manufacturer, is not guaranteed or endorsed by the publisher.
